# Vitality and Challenging Commitment in Times of Digital Intensification: Evidence for Healthy Educational Organizations Based on Teacher Engagement in Chile

**DOI:** 10.3390/ejihpe16030044

**Published:** 2026-03-13

**Authors:** Eduardo Sandoval-Obando, Stephanie Armstrong-Gallegos, Mauricio Véliz-Campos, Guido Salazar-Sepúlveda, Alejandro Vega-Muñoz, Miguel Salazar-Muñoz

**Affiliations:** 1 Escuela de Psicología, Facultad de Ciencias Sociales y Humanidades, Instituto Iberoamericano de Desarrollo Sostenible (IIDS), Universidad Autónoma de Chile, Temuco 4800916, Chile; 2 Institute of ICT in Education, Universidad de La Frontera, Temuco 4811322, Chile; 3 Facultad de Educación, Universidad de Talca, Talca 3460000, Chile; 4 Facultad de Ingeniería, Universidad Católica de la Santísima Concepción, Concepción 4090541, Chile; 5 Facultad de Ingeniería y Negocios, Universidad de Las Américas, Concepción 4090940, Chile; 6 Laboratorio de Bienestar y Comportamiento Organizacional, Universidad Central de Chile, Santiago 8330507, Chile; 7 Facultad de Ciencias Empresariales, Universidad Arturo Prat, Santiago 8340232, Chile; 8 Faculty of Psychology, Universidad San Sebastián, Puerto Montt 5480000, Chile; miguel.salazar@uss.cl

**Keywords:** work engagement, teacher well-being, digital intensification, occupational health, educational inequalities, Chilean teachers

## Abstract

The rapid digital transformation of education systems has profoundly changed teachers’ working conditions, intensified administrative demands, and highlighted territorial and organizational inequalities. In this context, understanding how these dynamics influence teacher engagement is essential for promoting healthy educational organizations. This study examined the factor structure of the UWES-17 and analyzed the relationship between engagement levels and sociodemographic variables in a sample of 314 elementary school teachers from four regions of Chile. Descriptive analyses, exploratory factor analysis with polychoric correlations and unweighted least squares, and confirmatory factor analysis using robust ULS and the Hull method were performed. The results showed a robust two-factor structure—Inspired Vitality and Challenging Commitment—with excellent fit indices. Freeman–Halton exact tests showed that Inspired Vitality was significantly associated with age, gender, region, location, administrative dependency, and professional experience, while Challenging Commitment was associated with gender, region, context, and professional experience. These findings indicate that teacher engagement is influenced by both structural inequalities and individual trajectories. The results underscore the need to strengthen organizational resources, regulate digital intensification, and reduce territorial gaps to promote teacher well-being.

## 1. Introduction

The accelerated digital transformation of education systems (Lynch et al., 2024; Samala et al., 2024), intensified by the COVID-19 pandemic, has profoundly reshaped the social and working conditions of teachers (Akour & Alenezi, 2022; Røe et al., 2022; Deroncele-Acosta et al., 2023; Bitar & Davidovich, 2024; C. Wang et al., 2024; Zhai, 2025). The expansion of hybrid modalities, the increase in technology-mediated administrative tasks, and the blurring of boundaries between work and personal life have generated new psychosocial stresses that affect teachers’ occupational health (Bacova & Turner, 2023; Siddiqui et al., 2023; Hussain et al., 2024; Lillelien & Jensen, 2025), and the functioning of healthy educational organizations (Akour & Alenezi, 2022; Gulliksen et al., 2023). At the same time, digital tools have also enabled new forms of pedagogical communication, reduced repetitive tasks, and expanded opportunities for instructional innovation, illustrating that technology can operate not only as a source of demand but also as a meaningful resource for teachers.

In this sense, the dual role of technology, as both a potential stressor and a potential support, aligns with the Job Demands–Resources (JD-R) model, which posits that well-being and performance depend on the balance between job demands and the personal and organizational resources available to workers (W. Schaufeli & Bakker, 2003; W. Schaufeli, 2021). When digital systems are implemented without adequate training, infrastructure, or institutional support, they may intensify workload and contribute to technostress. Conversely, when supported by appropriate conditions, digital tools can enhance autonomy, streamline administrative processes, and even strengthen work engagement.

In Latin America, these transformations are expressed unevenly across administrative dependence, territorial contexts, and institutional conditions, deepening digital divides and differences in teachers’ workloads (Bruggeman et al., 2022; Van De Werfhorst et al., 2022; Weber et al., 2025). In schools serving socially vulnerable communities, the rapid incorporation of educational platforms has expanded the repertoire of teaching tasks, often without sufficient technical or pedagogical support, increasing pressure for constant availability and exposure to fragmented information flows (Ahiaku et al., 2025; Cone et al., 2022; Luo et al., 2022; Pavez et al., 2024). These dynamics have contributed to rising digital overload and technostress (Zhong & Rosli, 2025), with documented effects on teachers’ well-being and mental health (Fiore & Decataldo, 2022; Rahmi et al., 2025; Sobral et al., 2025; Yang et al., 2023; K. Wang et al., 2025; Willermark et al., 2023). Such inequalities underscore the need to understand how territorial and organizational conditions shape teachers’ experiences of engagement in digitally intensified environments.

Within this context, work engagement emerges as a key motivational resource. In education, engagement is associated with energy, resilience, pedagogical meaning, and job satisfaction (Greenier et al., 2021; E. E. Sandoval-Obando et al., 2023; E. Sandoval-Obando et al., 2025). However, the relationship between digital workload, technostress, and teacher engagement remains an emerging field (Yang et al., 2023; Xing, 2022; K. Wang et al., 2025; A. Zhang & Yang, 2021), particularly in settings where technological and organizational inequalities may amplify tensions and produce divergent patterns of commitment.

In Chile, where territorial disparities and structural differences between municipal and subsidized private schools persist, there is a pressing need for empirical evidence that characterizes socio-labor conditions, digital workload, and levels of teacher engagement across diverse educational contexts. The purpose of this study was to describe these conditions and evaluate the factorial structure of the UWES-17 in elementary school teachers from four regions of the country, providing contextualized evidence for the design of healthy educational organizations in times of digital intensification.

## 2. Materials and Methods

The study adopted a quantitative, non-experimental, cross-sectional design aimed at evaluating the factorial structure of the UWES-17 (Occupational Health Psychology Unit Utrecht University, 2011) and describing the levels of teacher engagement among elementary school teachers in four regions of Chile. The stratified sample (Song & Kawai, 2023) consisted of 314 teachers from municipal and subsidized private schools in the Metropolitan, Maule, La Araucanía, and Los Ríos regions of Chile, including both urban and rural schools, with diverse professional experience and academic backgrounds. Although stratification ensured representation across regions and school types, the sampling procedure was non-probabilistic and based on voluntary participation, which aligns with common practices in occupational health research involving school systems. Specifically, the following inclusion criteria were considered: (i) holding a professional degree in primary education; (ii) working in primary education; (iii) carrying out their educational work in school settings (urban or rural) and in schools (municipal or subsidized private); and (iv) having three or more years of classroom teaching experience. Challenges associated with non-probabilistic sampling in educational research were considered, following the methodological recommendations of Kanaki and Kalogiannakis (2023). The response rate could not be calculated precisely because schools disseminated the survey internally; however, no systematic differences were detected between participating and non-participating schools based on available administrative information. Data collection was conducted using a self-administered, anonymous, voluntary digital questionnaire after obtaining informed consent.

The study complied with the ethical principles of the Declaration of Helsinki and was also approved by the scientific research ethics committee of the Autonomous University of Chile, according to resolution No. 11–25 of 22 April 2025.

The instrument used was the Utrecht Work Engagement Scale (UWES-17), which has been widely validated in various work contexts (W. Schaufeli & Bakker, 2003; W. B. Schaufeli et al., 2009; W. B. Schaufeli & Taris, 2014). The UWES-17 assesses engagement through 17 ordinal items which, in their original formulation, are organized into three dimensions: Vigor, Dedication, and Absorption. Given that previous research has shown factorial variations across cultural and occupational contexts, it was considered relevant to examine its structure among Chilean teachers.

Data analysis was carried out in several stages. First, a preliminary examination of the items was conducted using univariate descriptive statistics (mean, variance, skewness, and kurtosis), in accordance with psychometric criteria for ordinal distributions (Ferrando et al., 2022; Ho & Yu, 2015). Sample adequacy was assessed using the sample adequacy measure (SAM) based on the anti-image matrix (Kaiser & Cerny, 1979), the KMO index, and Bartlett’s sphericity test, confirming the relevance of applying factor analysis.

Subsequently, exploratory factor analysis (EFA) was performed using polychoric correlations, the Unweighted Least Squares (ULS) extraction method, and Direct Oblimin oblique rotation with Kaiser normalization. ULS was selected because it performs well with ordinal data, does not assume multivariate normality, and is robust with moderate sample sizes—conditions that characterize the present dataset, as recommended by Krijnen (1996), Ximénez and García (2005), Li (2016), and Morata-Ramírez et al. (2015). EFA allowed us to identify the data’s underlying structure as a preliminary step toward confirmatory analysis.

Next, a confirmatory factor analysis (CFA) was performed using FACTOR software version 12.01.02 of December 2021 (Rovira i Virgili University, Tarragona, Spain) (Ferrando & Lorenzo-Seva, 2017), employing polyarchic correlations, the Robust Unweighted Least Squares (RULS) method, and factor selection using the Hull method (Lorenzo-Seva et al., 2011). This combination of procedures is recommended for ordinal items and avoids the inflation of fit indices that can occur with maximum likelihood estimation in non-normal data. Model fit was evaluated using the indices recommended by Schermelleh-Engel et al. (2003), including: Chi-square divided by degrees of freedom (χ^2^/df), Root Mean Square Error of Approximation (RMSEA), Adjusted Goodness of Fit Index (AGFI), Goodness of Fit Index (GFI), Comparative Fit Index (CFI), Non-Normed Fit Index (NNFI), Root Mean Square Residual (RMSR), following the interpretation criteria proposed by Kalkan and Kelecioğlu (2016). The internal reliability of the resulting factors was estimated using Cronbach’s alpha coefficient, following the recommendations of Bonett and Wright (2015).

Finally, to analyze the relationship between engagement levels and the sociodemographic characteristics of teachers (age, gender, region, location, administrative dependence, professional experience, and educational level), the exact Freeman–Halton test was applied, an extension of Fisher’s exact test for RxC tables (Freeman & Halton, 1951). This procedure is particularly suitable when expected frequencies are low and maintains the probabilistic accuracy of the exact test, as recommended by Contreras-Cristán and González-Barrios (2009) and Ruxton and Neuhäuser (2010). The calculation was performed using the exact tests option in SPSS version 23 (IBM, New York, NY, USA), which automatically applies this extension to larger contingency tables.

## 3. Results

The sample characterization showed a heterogeneous distribution across age, gender, professional experience, educational level, administrative dependence, and geographical location. Participants included teachers from municipal (public) and subsidized private (mixed funding) schools located in both urban and rural areas of the Metropolitan, Maule, La Araucanía, and Los Ríos regions. This diversity allowed the analysis of engagement patterns across contrasting territorial and organizational conditions, providing a broad view of the Chilean elementary school context (Table 1).

### 3.1. Univariate Item Analysis

The 17 items of the UWES-17 showed high means (3.90–4.74), indicating elevated engagement levels in the sample. Variances were adequate, and no items presented insufficient dispersion. Distributions showed moderate negative skewness and acceptable kurtosis values for ordinal data. These patterns supported the use of polychoric correlations and robust estimation methods, as they reduce bias when items deviate from normality (Table 2).

### 3.2. Exploratory Factor Analysis

Exploratory factor analysis (EFA) revealed a two-factor structure, in contrast to the classic three-factor structure of the UWES-17. The first factor grouped items related to energy, enthusiasm, meaning, and positive absorption, while the second factor grouped items associated with persistence, intense absorption, and difficulty disconnecting. This configuration suggests two distinct but related engagement profiles among Chilean teachers.

The first factor explained more than half of the total variance, while the second factor contributed an additional meaningful percentage. Communalities were high for most items, and the sample adequacy indices (KMO and MSA) confirmed the suitability of the factor model (Table 3).

**Figure 1:**
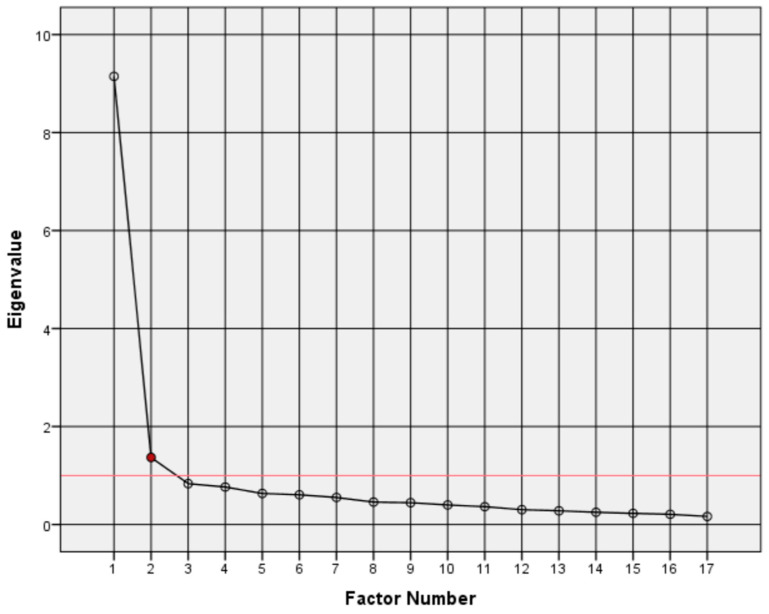
Scree plot. Elbow point indicated in red and clipping line (eigenvalue = 1) in red line.

Figure 1 shows the scree plot, where the elbow point (eigenvalue = 1.368) coincides with the clipping line (eigenvalue = 1), supporting the retention of two factors.

### 3.3. Confirmatory Factor Analysis

Confirmatory factor analysis (CFA) corroborated the two-factor structure identified in the EFA. The two-factor model showed excellent fit indices, with CFI and NNFI values close to 1.000, RMSEA equal to 0.000, and GFI and AGFI greater than 0.99. In contrast, the unifactorial model showed weaker fit across all indices. The difference between the EFA eigenvalue for Factors and the CFA model-implied eigenvalue is expected because the two procedures rely on different estimation frameworks. EFA eigenvalues are extracted from the unrotated correlation matrix using ULS, whereas CFA eigenvalues are derived from the fitted RULS model and reflect the variance explained under confirmatory constraints. Therefore, eigenvalues from EFA and CFA are not directly comparable.

Factor loadings were high and consistent with the proposed structure, and internal reliability reached satisfactory levels for both factors (Table 4 and Table 5). The correlation between factors was substantial but not redundant, indicating related but distinct dimensions of engagement.

In Table 5, the internal consistency was evaluated using Cronbach’s alpha with 95% confidence intervals. Inspired Vitality showed excellent reliability (α = 0.942, 95% CI [0.936, 0.948]), while Challenging Commitment showed acceptable reliability (α = 0.795, 95% CI [0.772, 0.818]).

To facilitate interpretation for readers unfamiliar with psychometric modeling, a concise model comparison table was added, including the most relevant indices and the thresholds adopted (Table 6).

The two-factor model clearly outperformed the unifactorial alternative, supporting the conceptual distinction between Inspired Vitality and Challenging Commitment.

### 3.4. Associations with Sociodemographic Variables

The Freeman–Halton exact test revealed differentiated association patterns between engagement factors and sociodemographic variables.

- Inspired Vitality showed significant associations with age, gender, region, location, administrative dependence, and professional experience, but not with educational level.
- Challenging Commitment was significantly associated with gender, region, location, and experience, but not with age, administrative dependence, or educational level.
- The total UWES-17 score showed significant associations with all variables except educational level.

These results indicate that teacher engagement is shaped by both individual trajectories (e.g., experience, gender) and structural conditions (e.g., region, school location, administrative dependence), reinforcing the relevance of territorial and organizational inequalities in shaping teachers’ motivational resources. To complement statistical significance, effect sizes were calculated using Cramer’s V. Across all associations, effect sizes ranged from small to small-to-moderate (V = 0.115–0.288), indicating limited to modest practical relevance despite statistically significant results (Table 7).

## 4. Discussion

The results of this study provide robust evidence on the configuration of teacher engagement in the Chilean school context, identifying a two-factor structure of the UWES-17: Inspired Vitality and Challenging Commitment. This alternative structure differs from the classic three-factor model (W. Schaufeli & Bakker, 2003) and coincides with research indicating that, in vocational and emotionally demanding professions, the dimensions of vigor, dedication, and absorption tend to be integrated into broader or more intense patterns of work engagement (Rathan et al., 2025; Yuan et al., 2025). Among Chilean teachers, this bifurcation appears to reflect two distinct modalities of commitment shaped by digital intensification, administrative overload, and persistent territorial and historical-cultural inequalities.

Inspired Vitality brings together energy, enthusiasm, meaning, and positive absorption, suggesting a deep pedagogical involvement consistent with evidence on the role of intrinsic motivation, self-efficacy, and professional identity in teacher well-being (Greenier et al., 2021; Yuan et al., 2025). In contrast, Challenging Commitment captures a more demanding form of engagement characterized by persistence, intense absorption, and difficulty disconnecting, a pattern observed in high-demand professions, where intense involvement can coexist with risks of burnout when recovery is insufficient (Fujikawa et al., 2024; H. Zhang & Cao, 2025). Rather than interpreting this factor as inherently maladaptive, the present findings suggest that it may represent a culturally embedded form of professional dedication, particularly salient in contexts where teachers compensate for structural deficiencies through personal effort.

Based on the reported reliability coefficients and association patterns (see Table 6 and Table 7), it is possible to propose a socio-pedagogical analysis focused on the differential stability of teacher engagement patterns. The high internal consistency of Inspired Vitality and its strong association with structural variables indicate that this factor is sensitive to organizational resources, perceived fairness, and accumulated professional capital. In contrast, the moderate reliability of the weaker structural dependence of Challenging Commitment suggests a more transversal configuration, potentially rooted in vocational dispositions, coping strategies, or cultural norms that legitimize persistence under pressure. From this perspective, teachers’ engagement reflects not only objective working conditions but also professional socialization processes that shape how teachers interpret and respond to demands. Structural inequalities do not determine whether commitment exists, but they influence their pedagogical quality, sustainability, and long-term consequences (Delgado-Galindo et al., 2025; Holst et al., 2025; Martin & Benedetti, 2025; Zorde & Lapidot-Lefler, 2025).

The identification of the Challenging Commitment factor suggests the need to differentiate between adaptive and potentially dysfunctional forms of teacher engagement. Although its empirical indicators—high persistence, intense concentration on the task, and difficulty disconnecting from work—show partial convergence with constructs such as overcommitment (Siegrist & Li, 2016; Jin et al., 2025), heavy work investment (W. B. Schaufeli, 2016; Tabak et al., 2021; Wettstein et al., 2022), and strain-based involvement (Muasya, 2024; Provido et al., 2025; Sharif, 2025), the results of this study reveal a specific and differentiating configuration within the field of teacher engagement. Unlike excessive commitment associated with occupational stress—characterized by heightened physiological reactivity, progressive exhaustion, and declines in well-being—Challenging Commitment appears as a form of professional involvement oriented toward sustaining pedagogical continuity in highly demanding institutional contexts. From the perspective of the Job Demands–Resources model, this pattern may represent an adaptive response to workload intensification and organizational constraints. However, the difficulty in disconnecting suggests a blurred boundary between professional vocation and over-investment in work (Bredehorst et al., 2024). Consequently, Challenging Commitment reflects an ambivalent form of engagement that combines professional energy with the risk of cognitive overload, particularly in educational systems experiencing increasing institutional pressures and the digitalization of teaching work.

Sociodemographic analyses using the Freeman–Halton exact test show that Inspired Vitality is highly sensitive to structural inequalities, as it is associated with age, gender, region, location, administrative dependence, and professional experience. This pattern reinforces the idea that vitality and meaning in work depend strongly on organizational resources, consistent with the Job Demands–Resources (JD-R) model and with evidence linking engagement to institutional conditions, leadership, and professional development opportunities (Collie, 2023; Jamal et al., 2023; Timotheou et al., 2023; Cai et al., 2022; Polatbekova et al., 2025; Rathan et al., 2025). In contrast, Challenging Engagement showed greater stability and weaker dependence on structural variables, suggesting that it may reflect personal styles of involvement, emotion-focused coping, or traits such as tolerance for ambiguity, positively associated with engagement in clinical and educational contexts (Fujikawa et al., 2024).

However, the findings also point to the need for further investigation into the coexistence of functional and dysfunctional engagement in digital intensification. High engagement in contexts of technological overload and unequal institutional support (connectivity, and infrastructure) may constitute short-term adaptive responses but may be difficult to sustain. Challenging Commitment may reflect a normative internalization of self-imposed demands, where persistence and difficulty disconnecting are framed as part of the teaching vocation (Saks et al., 2022). Without adequate organizational resources and explicit regulation of digital workload, this configuration may increase vulnerability to progressive burnout.

For this reason, a dialogical perspective on technology is essential. Technology is not inherently a risk factor; rather, its impact depends on how it is integrated into organizational and pedagogical practices (Digón-Regueiro et al., 2023; Perrotta, 2013; Torres-Rivera et al., 2025; Ubal Camacho et al., 2023; Yan et al., 2025). According to the Job Demands-Resources (JD-R) model, digital tools can serve as work resources by optimizing planning, automating evaluation processes, and facilitating asynchronous communication, thereby reducing administrative burden and freeing up pedagogical time.

In schools with strong leadership and clear availability policies, technology can even support teacher disconnection, for example, through staggered scheduling platforms or notification-limiting systems. The problem arises when digitalization increases demand without strengthening resources. Thus, the key issue is not technology itself but the structural conditions that determine whether it becomes a protective resource or a chronic demand.

The results are consistent with evidence showing that emotional intelligence, self-efficacy, and emotional regulation predict teacher engagement (Deng et al., 2022; Hameli et al., 2025; Uzuntiryaki-Kondakci et al., 2022; Yuan et al., 2025). Engagement is strengthened when personal and organizational resources are available to manage emotional and cognitive demands in contexts of uncertainty or digital transformation (Harper-Hill et al., 2022; Polatbekova et al., 2025; Rathan et al., 2025). In Chile, the unequal distribution of these resources across regions, administrative types, and urban/rural contexts limits the capacity to sustain high levels of vitality and meaning in work (Ahiaku et al., 2025; Iturra & Gallardo, 2022).

To illustrate this, consider two contrasting scenarios. In an urban school with stable technological support and distributed leadership, a teacher with ten years of experience may experience high Inspired Vitality, using digital platforms for efficient feedback and professional collaboration, which strengthens their sense of purpose and work energy. In a rural school with unreliable connectivity and limited institutional support, a teacher may exhibit high Challenging Commitment, characterized by persistence and prolonged hyper-connectivity to compensate for structural deficiencies. Both profiles demonstrate commitment, but their functional configurations differ, showing how similar digital demands can lead to healthy engagement or sustained overexertion depending on the balance between demands and resources.

### 4.1. Limitations

This study has some limitations that should be considered when interpreting the results. First, cross-sectional design prevents establishing causal relationships between the variables analyzed, so future research should incorporate longitudinal designs to examine the evolution of engagement over time. Second, the data are based on self-reports, which may introduce social desirability or subjective perception biases, a common limitation in occupational well-being and health studies (W. Schaufeli, 2021). Third, although the sample is geographically diverse, it does not include private paid establishments or extreme regions, which limits the generalizability of the findings. Additionally, the voluntary nature of participation may have introduced self-selection bias, and the absence of information on non-respondents restricts the assessment of potential systematic differences. Finally, the study focused on the UWES-17; recent research suggests exploring alternative or complementary scales, such as the Engaged Teacher Scale (ETS) or culturally adapted versions (Rathan et al., 2025).

### 4.2. Organizational and Socio-Pedagogical Implications

Strengthening labor resources is essential for sustaining the Inspired Vitality of teachers, particularly in public and rural schools where structural constraints may weaken teachers’ sense of effectiveness and recognition. Pedagogical leadership emphasizes professional support, emotional accompaniment, and systematic recognition can enhance engagement, alongside administrative simplification strategies that protect pedagogical time. Regulating digital intensification is also key to preventing Challenging Commitment configurations associated with persistence under pressure and difficulty disconnecting. Institutional policies on availability, communication protocols, and training in time management and the functional use of technologies can mitigate these risks (Mexhuani, 2025). Such actions should be adapted to urban or rural contexts and administrative dependency to reduce regional gaps and ensure minimum standards of teacher well-being.

From a pedagogical perspective, integrating engagement into professional development allows continuous training to focus on skills that expand personal and collective resources in the face of growing demands (Moravec & Martínez-Bravo, 2023). Programs that strengthen self-efficacy, professional autonomy, and emotional intelligence promote emotional regulation and teacher dedication in contexts of uncertainty, while initiatives that foster creativity, flexibility, and tolerance for ambiguity facilitate adaptation to technological and organizational changes (Rennstich, 2023; Tomczyk & Majkut, 2025). These guidelines must align with educational policies aimed at psychological well-being, resilience, and continuous teacher development (Shu, 2022; Holzer & Spiel, 2025; Ma & Pongpisanu, 2025). Finally, future lines of research should incorporate longitudinal, comparative, and mixed-methods approaches, integrating variables such as generativity (E. E. Sandoval-Obando et al., 2023; E. Sandoval-Obando et al., 2025), self-efficacy and emotional intelligence (Xiao et al., 2022), and the evaluation of successful organizational interventions (Polatbekova et al., 2025).

## 5. Conclusions

The study provides contextualized and psychometrically robust evidence on teacher engagement in the Chilean elementary schools, identifying a two-factor structure of the UWES-17 composed of Inspired Vitality and Challenging Commitment. This bifactorial configuration offers a more nuanced understanding of engagement in digitally intensified educational environments, where teachers navigate increasing administrative demands, uneven technological conditions, and persistent territorial inequalities.

Inspired Vitality emerged as a resource-sensitive dimension, strongly associated with structural and organizational variables such as region, school location, administrative dependence, and professional experience. This suggests that teachers’ energy, meaning, and positive absorption are closely linked to the availability of institutional support, technological infrastructure, and equitable working conditions. In contrast, Challenging Commitment reflected a more stable and transversal pattern, less dependent on structural factors and potentially rooted in vocational dispositions, coping strategies, and cultural norms that legitimize persistence under pressure.

Together, these findings highlight that engagement is not a uniform construct, but rather a dynamic interplay between personal resources and contextual demands. In settings where digital intensification is accompanied by adequate organizational support, engagement may foster well-being and pedagogical effectiveness. However, in contexts marked by technological overload, limited institutional resources, or territorial disparities, engagement, particularly in its challenging form, may become difficult to sustain, increasing vulnerability to burnout.

The study underscores the importance of strengthening organizational resources, regulating digital workload, and reducing territorial gaps to promote healthy and sustainable forms of engagement. It also points to the need for professional development initiatives that enhance emotional regulation, self-efficacy, and adaptive coping in the face of technological and organizational change.

Finally, the results open avenues for future research, including longitudinal analyses of engagement trajectories, comparative studies across school types and regions, and the integration of complementary constructs such as generativity, emotional intelligence, and organizational justice. Such approaches would deepen understanding of how teachers maintain vitality and commitment in increasingly complex educational environments.

## Figures and Tables

**Table 1:** Teachers sample characterization.

Sociodemographic Variables	Level (Number Code)	*n*	*n*%
Gender (GND)	Female (1) *	242	77.1%
	Male (2)	70	22.3%
	No binary (3)	2	0.6%
Age level (AGE) (in years)	AGL < 30 (1)	47	15.0%
	30 ≤ AGL ≤ 39 (2)	104	33.1%
	40 ≤ AGL ≤ 49 (3)	100	31.8%
	50 ≤ AGL ≤ 59 (4)	41	13.1%
	60 ≤ AGL (5)	22	7.0%
Region (REG)	La Araucanía (LAR)	102	32.5%
	Los Ríos (LRI)	70	22.3%
	Maule (MAU)	80	25.5%
	Metropolitan (MET)	62	19.7%
Location (LOC)	Rural (1)	94	29.9%
	Urban (2)	220	70.1%
Dependence (DEP)	Public (1)	121	38.5%
	Mixed funding (2)	193	61.5%
Experience level (EXP) (in years)	EXP < 5 (1)	41	13.1%
	5 ≤ EXP ≤ 10 (2)	100	31.8%
	11 ≤ EXP ≤ 15 (3)	85	27.1%
	16 ≤ EXP ≤ 20 (4)	39	12.4%
	21 ≤ EXP (5)	49	15.6%
Education level (EDU)	Ungraduated (1) **	227	72.3%
	Qualification (2) ***	33	10.5%
	Advanced qualification (3)	5	1.6%
	Master (4)	49	15.6%

* The numbers and acronyms in parentheses (°) correspond to the values assigned in the database. ** Ungraduated includes teachers who have not completed postgraduate studies. *** Qualification, including teachers with varying levels of pedagogical specialization at the diploma and postgraduate levels in educational fields.

**Table 2:** Univariate descriptive statistical analysis.

Variables	N	Mean	Variance	Skewness		Kurtosis	
	Statistic	Statistic	Statistic	Statistic	Std. Error	Statistic	Std. Error
WBW1 *	314	4.10	2.188	−0.630	0.138	−0.454	0.274
WBW2 *	314	4.58	2.027	−0.901	0.138	0.141	0.274
WBW3 *	314	4.49	2.136	−0.899	0.138	0.044	0.274
WBW4 *	314	4.31	2.171	−0.762	0.138	−0.262	0.274
WBW5 *	314	4.43	2.323	−1.013	0.138	0.428	0.274
WBW6 *	314	3.91	2.972	−0.673	0.138	−0.411	0.274
WBW7 *	314	4.54	2.147	−0.992	0.138	0.322	0.274
WBW8 *	314	3.90	2.884	−0.588	0.138	−0.594	0.274
WBW9 *	314	4.06	2.645	−0.645	0.138	−0.498	0.274
WBW10 *	314	4.74	1.975	−1.188	0.138	0.883	0.274
WBW11 *	314	4.49	1.803	−0.748	0.138	−0.156	0.274
WBW12 *	314	3.99	2.377	−0.600	0.138	−0.369	0.274
WBW13 *	314	4.68	1.855	−1.350	0.138	1.835	0.274
WBW14 *	314	4.08	2.316	−0.797	0.138	0.325	0.274
WBW15 *	314	4.69	1.890	−1.236	0.138	1.359	0.274
WBW16 *	314	4.03	2.763	−0.752	0.138	−0.305	0.274
WBW17 *	314	4.40	2.491	−1.050	0.138	0.485	0.274
Valid N (listwise)	314						

* Variables that satisfy the pre-established parameters of standard deviation, skewness, and kurtosis.

**Table 3:** Exploratory factor analysis for two factors.

KMO and Bartlett’s Test				
Kaiser–Meyer–Olkin Measure of Sampling Adequacy				0.945
Bartlett’s Test of Sphericity		Approx. Chi-Square		3590.706
		Degree of freedom		136
		Significance		0.000
Pattern Matrix ^a^				
ID	Factor 1 (F1)		Factor 2 (F2)	
WBW1	0.792			
WBW2	0.780			
WBW3	0.699			
WBW4	0.703			
WBW5	0.847			
WBW6	0.471			
WBW7	0.942			
WBW8	0.870			
WBW9	0.827			
WBW10	0.696			
WBW11	0.508			
WBW12	0.510			
WBW13			0.450	
WBW14			0.490	
WBW15			0.519	
WBW16			0.634	
WBW17			0.691	
Eigenvalue	8.746		0.878	
% of Variance	51.450		5.162	
Cumulative %	51.450		56.611	
Factor Correlation Matrix ^b^				
Factor	1	2		
1	1.000	0.618		
2	0.618	1.000		

^a^ Extraction Method: Unweighted Least Squares. Rotation Method: Oblimin with Kaiser Normalization. Rotation converged in 6 iterations. ^b^ Extraction Method: Unweighted Least Squares. Rotation Method: Oblimin with Kaiser Normalization.

**Table 4:** Confirmatory factor analysis for two factors.

KMO and Bartlett’s Test			
Kaiser–Meyer–Olkin Measure of Sampling Adequacy (confidence interval 90%)			0.944 (0.855; 0.933)
Bartlett’s Test of Sphericity	Approx. Chi-Square		3528.7
	Degree of freedom		136
	Significance		0.000010
Rotated Loading Matrix			
Variable (Item)	Factor 1 (F1)	Factor 2 (F2)	
WBW1	0.850		
WBW2	0.831		
WBW3	0.734		
WBW4	0.762		
WBW5	0.936		
WBW6	0.554		
WBW7	0.969		
WBW8	0.879		
WBW9	0.810		
WBW10	0.663		
WBW11	0.470		
WBW12	0.506		
WBW13		0.548	
WBW14		0.598	
WBW15		0.576	
WBW16		0.721	
WBW17		0.723	
Explained Variance	0.602	0.081	
Cumulative Variance	0.602	0.683	
Eigenvalue	10.234	1.368	
Inter Factor Correlation Matrix			
Factor	F1	F2	
F1	1.000		
F2	0.665	1.000	

**Table 5:** Reliability statistics.

Scale	Variance	Skewness	Kurtosis	Valid Cases	Number of Items	Cronbach’s Alpha
Factor 1	1.567	−0.431	−0.704	314	12	0.942 ci (0.936 0.948) **
Factor 2	1.380	−0.332	−0.683	314	5	0.795 ci (0.772 0.818) *
Factor Total	1.341	−0.377	−0.626	314	17	0.942 ci (0.936 0.948) **

* Cronbach’s Alpha > 0.7, ** Cronbach’s Alpha > 0.8.

**Table 6:** Validation and reliability versus parameters (Schermelleh-Engel et al., 2003).

Model	Sample	Level	Cronbach’s Alpha	MIF	χ^2^/df	RMSEA	AGFI	GFI	CFI	NNFI	RMSR
Proposed (Two-factor model)	314	-	0.934 **	5	0.54 **^,+^	0.000 ** ci (could not be computed)	0.996 ** ci (0.996 0.997)	0.997 ** ci (0.997 0.998)	0.999 ** ci (0.998 1.001)	1.000 ** ci (1.001 1.002)	0.036 ** ci (0.033 0.035)
Contrast (One-factor model)	314	-	0.934 **	17	1.52 ** ^+^	0.072 * ci (0.057 0.078)	0.988 ** ci (0.986 0.992)	0.989 ** ci (0.987 0.993)	0.987 ** ci (0.983 0.993)	0.985 ** ci (0.980 0.992)	0.065 ** ci (0.057 0.069)
Adopted thresholds	≥200	**	[0.80, 0.95)	NR	[0, 2]	[0.00, 0.05]	[0.90, 1.00]	[0.95, 1.00]	[0.97, 1.00]	[0.97, 1.00]	[0.00, 0.05) ^++^
		*	[0.70, 0.80)	≥3	(2, 3]	(0.05, 0.08]	[0.85, 0.90)	[0.90, 0.95)	[0.95, 0.97)	[0.95, 0.97)	[0.05, 0.08] ^++^

NR: not reported. ** Good fit. * Acceptable fit. ^+^ Minimum Fit Function Chi Square. ^++^ indicated in Kalkan and Kelecioğlu (2016).

**Table 7:** Freeman–Halton (F-H) extension of Fisher’s Exact Tests.

Variable 1	Variable 2	N of Valid Cases	Value F-H ^+^	Significance (2-Sided) ^+^	Correlation Evidence	Cramer’s V	Effect Size
F1	AGE	314	53.203	0.000 ci (0.000 0.000) **	Yes	0.213	small–moderate
	GND	314	26.024	0.001 ci (0.000 0.002) **	Yes	0.210	small–moderate
	REG	314	49.511	0.000 ci (0.000 0.000) **	Yes	0.242	small–moderate
	LOC	314	24.231	0.000 ci (0.000 0.000) **	Yes	0.280	small–moderate (close to medium)
	DEP	314	12.145	0.025 ci (0.021 0.029) *	Yes	0.197	small
	EXP	314	37.204	0.005 ci (0.003 0.006) **	Yes	0.172	small
	EDU	314	19.136	0.172 ci (0.162 0.182)	No	0.139	small
F2	AGE	314	26.833	0.109 ci (0.101 0.117)	No	0.145	small
	GND	314	26.762	0.001 ci (0.000 0.001) **	Yes	0.192	small
	REG	314	46.122	0.000 ci (0.000 0.000) **	Yes	0.227	small–moderate
	LOC	314	12.869	0.018 ci (0.014 0.021) *	Yes	0.207	small–moderate
	DEP	314	9.931	0.062 ci (0.055 0.068)	No	0.180	small
	EXP	314	36.296	0.006 ci (0.004 0.008) **	Yes	0.170	small
	EDU	314	15.148	0.526 ci (0.513 0.539)	No	0.115	small
FT	AGE	314	49.420	0.000 ci (0.000 0.000) **	Yes	0.201	small–moderate
	GND	314	27.007	0.001 ci (0.000 0.002) **	Yes	0.208	small–moderate
	REG	314	57.286	0.000 ci (0.000 0.000) **	Yes	0.255	small–moderate
	LOC	314	24.679	0.000 ci (0.000 0.000) **	Yes	0.288	small–moderate (close to medium)
	DEP	314	24.722	0.000 ci (0.000 0.000) **	Yes	0.283	small–moderate (close to medium)
	EXP	314	39.784	0.002 ci (0.001 0.003) **	Yes	0.179	small
	EDU	314	22.624	0.081 ci (0.074 0.088)	No	0.147	small

* *p*-value < 0.05, ** *p*-value < 0.01, ^+^ calculated with Monte Carlo significance based on 10,000 sampled tables with starting seed 2,000,000.

## Data Availability

Anonymized data availability in Supplementary Materials.

## Supplementary Materials

The following supporting information can be downloaded at: https://www.mdpi.com/article/10.3390/ejihpe16030044/s1, Table S1: WBW_data.csv.

## Abbreviations

The following abbreviations are used in this manuscript:

AGFI	Adjusted Goodness of Fit Index
AGL	Age Level
CFA	Confirmatory Factor Analysis
CFI	Comparative Fit Index
df	Degrees of Freedom
EDL	Educational Level
EFA	Exploratory Factor Analysis
F1	Factor One
F2	Factor Two
FT	Factor Total
GFI	Goodness of Fit Index
GND	Gender
KMO	Kaiser–Meyer–Olkin
MIF	Minimal Number of Items per Factor
MSA	Measurement of Sampling Adequacy
MSA	Measure of Sampling Adequacy
NNFI	Non-Normed Fit Index
RMSEA	Root Mean Square Error of Approximation
RMSR	Root Mean Square of Residuals
RULS	Robust Unweighted Least Squares
SPSS	Statistical Package for the Social Sciences
TLI	Tucker & Lewis Index
ULS	Unweighted Least Squares
UWES	Utrecht Work Engagement Scale

## References

[B1-ejihpe-16-00044] Ahiaku P. K. A., Uleanya C., Muyambi G. C. (2025). Rural schools and tech use for sustainability: The challenge of disconnection. Education and Information Technologies.

[B2-ejihpe-16-00044] Akour M., Alenezi M. (2022). Higher education future in the era of digital transformation. Education Sciences.

[B3-ejihpe-16-00044] Bacova D., Turner A. (2023). Teacher vulnerability in teacher identity in times of unexpected social change. Research in Post-Compulsory Education.

[B4-ejihpe-16-00044] Bitar N., Davidovich N. (2024). Transforming pedagogy: The digital revolution in higher education. Education Sciences.

[B5-ejihpe-16-00044] Bonett D. G., Wright T. A. (2015). Cronbach’s alpha reliability: Interval estimation, hypothesis testing, and sample size planning. Journal of Organizational Behavior.

[B6-ejihpe-16-00044] Bredehorst J., Krautter K., Meuris J., Jachimowicz J. M. (2024). The challenge of maintaining passion for work over time: A daily perspective on passion and emotional exhaustion. Organization Science.

[B7-ejihpe-16-00044] Bruggeman B., Garone A., Struyven K., Pynoo B., Tondeur J. (2022). Exploring university teachers’ online education during COVID-19: Tensions between enthusiasm and stress. Computers and Education Open.

[B8-ejihpe-16-00044] Cai Y., Wang L., Bi Y., Tang R. (2022). How can the professional community influence teachers’ work engagement? The mediating role of teacher self-efficacy. Sustainability.

[B9-ejihpe-16-00044] Collie R. J. (2023). Job demands and resources, teachers’ subjective vitality, and turnover intentions: An examination during COVID-19. Educational Psychology.

[B10-ejihpe-16-00044] Cone L., Brøgger K., Berghmans M., Decuypere M., Förschler A., Grimaldi E., Hartong S., Hillman T., Ideland M., Landri P., Van De Oudeweetering K., Player-Koro C., Bergviken Rensfeldt A., Rönnberg L., Taglietti D., Vanermen L. (2022). Pandemic Acceleration: Covid-19 and the emergency digitalization of European education. European Educational Research Journal.

[B11-ejihpe-16-00044] Contreras-Cristán A., González-Barrios J. M. (2009). A nonparametric test for symmetry based on freeman and halton’s ideas on contingency tables. Communications in Statistics—Simulation and Computation.

[B12-ejihpe-16-00044] Delgado-Galindo P., García-Jiménez J., Torres-Gordillo J.-J., Rodríguez-Santero J. (2025). School climate and academic performance: Key factors for sustainable education in high-efficacy schools and low-efficacy schools. Sustainability.

[B13-ejihpe-16-00044] Deng J., Heydarnejad T., Farhangi F., Farid Khafaga A. (2022). Delving into the relationship between teacher emotion regulation, self-efficacy, engagement, and anger: A focus on English as a foreign language teachers. Frontiers in Psychology.

[B14-ejihpe-16-00044] Deroncele-Acosta A., Palacios-Núñez M. L., Toribio-López A. (2023). Digital transformation and technological innovation on higher education post-COVID-19. Sustainability.

[B15-ejihpe-16-00044] Digón-Regueiro P., Gewerc-Barujel A., Pérez-Crego C. (2023). Dilemas en la integración de tecnologías en el aula de primaria: El diálogo entre la agencia docente, el currículo y las tecnologías digitales. Pedagogías: Revista Internacional.

[B16-ejihpe-16-00044] Ferrando P. J., Lorenzo-Seva U. (2017). Program FACTOR at 10: Origins, development and future directions. Psicothema.

[B17-ejihpe-16-00044] Ferrando P. J., Lorenzo-Seva U., Hernández-Dorado A., Muñiz J. (2022). Decalogue for the factor analysis of test items. Psicothema.

[B18-ejihpe-16-00044] Fiore B., Decataldo A. (2022). Digital-insecurity and overload: The role of technostress in lecturers’ work-family balance. Italian Journal of Sociology of Education.

[B19-ejihpe-16-00044] Freeman G. H., Halton J. H. (1951). Note on an exact treatment of contingency, goodness of fit and other problems of significance. Biometrika.

[B20-ejihpe-16-00044] Fujikawa H., Aoki T., Ando T., Haruta J. (2024). Associations of clinical context-specific ambiguity tolerance with burnout and work engagement among Japanese physicians: A nationwide cross-sectional study. BMC Medical Education.

[B21-ejihpe-16-00044] Greenier V., Derakhshan A., Fathi J. (2021). Emotion regulation and psychological well-being in teacher work engagement: A case of British and Iranian English language teachers. System.

[B22-ejihpe-16-00044] Gulliksen J., Lilliesköld J., Stenbom S. (2023). The ‘New’ new normal—Digitalization and hybridization of work and education before, during and after the COVID-19 pandemic. Interacting with Computers.

[B23-ejihpe-16-00044] Hameli K., Ukaj L., Çollaku L. (2025). The role of self-efficacy and psychological empowerment in explaining the relationship between emotional intelligence and work engagement. EuroMed Journal of Business.

[B24-ejihpe-16-00044] Harper-Hill K., Beamish W., Hay S., Whelan M., Kerr J., Zelenko O., Villalba C. (2022). Teacher engagement in professional learning: What makes the difference to teacher practice?. Studies in Continuing Education.

[B25-ejihpe-16-00044] Ho A. D., Yu C. C. (2015). Descriptive statistics for modern test score distributions: Skewness, kurtosis, dis-creteness, and ceiling effects. Educational and Psychological Measurement.

[B26-ejihpe-16-00044] Holst J., Brock A., Grund J., Schlieszus A.-K., Singer-Brodowski M. (2025). Whole-school sustainability at the core of quality education: Wished for by principals but requiring collective and structural action. Journal of Cleaner Production.

[B27-ejihpe-16-00044] Holzer J., Spiel C. (2025). Teachers’ occupational strain and subjective well-being: Digital teaching makes a difference. Teacher Development.

[B28-ejihpe-16-00044] Hussain Z., Chenmei C., Saeed M., Hassan N., Chiragh F. (2024). Personality and teachers’ burnout stress: Exploring the digital competence as personal job resource in allied health institutions. Frontiers in Psychology.

[B29-ejihpe-16-00044] Iturra V., Gallardo M. (2022). Schools, circumstances and inequality of opportunities in Chile. International Journal of Educational Development.

[B30-ejihpe-16-00044] Jamal A., Yaqoob S., Hussain T., Alam H. (2023). Impact of perceived organizational support on innovative work behavior and burn out in teachers: Thriving at work as the mediator (2020). Journal of Education and Educational Development.

[B31-ejihpe-16-00044] Jin M., Ji Y., Ding S., Wu X. (2025). The effects of dual job demands on primary and secondary school teachers’ subjective well-being: A moderated mediation model. Current Psychology.

[B32-ejihpe-16-00044] Kaiser H. F., Cerny B. A. (1979). Factor analysis of the image correlation matrix. Educational and Psy-Chological Measurement.

[B33-ejihpe-16-00044] Kalkan Ö. K., Kelecioğlu H. (2016). The effect of sample size on parametric and nonparametric factor analytical methods. Educational Sciences: Theory & Practice.

[B34-ejihpe-16-00044] Kanaki K., Kalogiannakis M. (2023). Sample design challenges: An educational research paradigm. International Journal of Technology Enhanced Learning.

[B35-ejihpe-16-00044] Krijnen W. P. (1996). Algorithms for unweighted least-squares factor analysis. Computational Statistics & Data Analysis.

[B36-ejihpe-16-00044] Li C.-H. (2016). The performance of ML, DWLS, and ULS estimation with robust corrections in structural equation models with ordinal variables. Psychological Methods.

[B37-ejihpe-16-00044] Lillelien K., Jensen M. T. (2025). Digital and digitized interventions for teachers’ professional well-being: A systematic review of work engagement and burnout using the job demands–resources theory. Education Sciences.

[B38-ejihpe-16-00044] Lorenzo-Seva U., Timmerman M. E., Kiers H. A. L. (2011). The hull method for selecting the number of common factors. Multivariate Behavioral Research.

[B39-ejihpe-16-00044] Luo H., Zuo M., Wang J. (2022). Promise and reality: Using ICTs to bridge China’s rural–urban divide in education. Educational Technology Research and Development.

[B40-ejihpe-16-00044] Lynch J., Auld G., O’Mara J., Cloonan A. (2024). Teachers’ everyday work-for-change: Implementing curriculum policy in ‘disadvantaged’ schools. Journal of Education Policy.

[B41-ejihpe-16-00044] Ma Y., Pongpisanu S. (2025). Resilience in the digital age: Technostress and its impact on university lecturers in China. Journal of Cultural Analysis and Social Change.

[B42-ejihpe-16-00044] Martin E. M., Benedetti C. (2025). Teacher retention in high-poverty urban schools: The role of empowerment, leadership, and collaboration. Education and Urban Society.

[B43-ejihpe-16-00044] Mexhuani B. (2025). Adopting digital tools in higher education: Opportunities, challenges and theoretical insights. European Journal of Education.

[B44-ejihpe-16-00044] Morata-Ramírez M. A., Holgado-Tello F. P., Barbero-García I., Méndez G. (2015). Confirmatory factor analysis: Recommendations for underweighted least squares method related to chi-square and RMSEA type I error. Ac-Ción Psicológica.

[B45-ejihpe-16-00044] Moravec J. W., Martínez-Bravo M. C. (2023). Global trends in disruptive technological change: Social and policy implications for education. On the Horizon: The International Journal of Learning Futures.

[B46-ejihpe-16-00044] Muasya G. (2024). The influence of family and work support in moderating work family conflict and emotional exhaustion among female urban public school teachers with young children in Kenya. Africa Education Review.

[B47-ejihpe-16-00044] Occupational Health Psychology Unit Utrecht University (2011). https://www.wilmarschaufeli.nl/publications/Schaufeli/Test%20Manuals/Test_manual_UWES_Espanol.pdf.

[B48-ejihpe-16-00044] Pavez I., Novoa-Echaurren A., Salinas-Layana A. (2024). Teachers’ situated knowledge: Addressing digital exclusion in rural contexts. Digital Education Review.

[B49-ejihpe-16-00044] Perrotta C. (2013). Do school-level factors influence the educational benefits of digital technology? A critical analysis of teachers’ perceptions. British Journal of Educational Technology.

[B50-ejihpe-16-00044] Polatbekova K., Yergubekova Z., Bostanova A., Shektibayev N., Abdigapbarova U. (2025). Development of the scientific and innovative potential of future teachers. Scientific Reports.

[B51-ejihpe-16-00044] Provido G., Quicho R. F., Ibarra F., Collantes L., Ibañez E., Mukminin A. (2025). Teachers motivation and work-family conflict: Perceptions of Fil-Am teachers. Acta Scientiarum. Education.

[B52-ejihpe-16-00044] Rahmi K. H., Fahrudin A., Supriyadi T., Herlina E., Rosilawati R., Ningrum S. R. (2025). Technostress and cognitive fatigue: Reducing digital strain for improved employee well-being: A literature review. Multidisciplinary Reviews.

[B53-ejihpe-16-00044] Rathan R., Kassab S. E., Schmidt H. G., Nederhand M., Woltman A. (2025). Teacher engagement in health profession education: A scoping review. BMC Medical Education.

[B54-ejihpe-16-00044] Rennstich J. K., Arnold M. (2023). Learning hybrid by doing hybrid: Teaching critical digital skills in a safe learning space. Handbook of applied teaching and learning in social work management education.

[B55-ejihpe-16-00044] Røe Y., Wojniusz S., Bjerke A. H. (2022). The digital transformation of higher education teaching: Four pedagogical prescritions to move active learning pedagogy forward. Frontiers in Education.

[B56-ejihpe-16-00044] Ruxton G. D., Neuhäuser M. (2010). Good practice in testing for an association in contingency tables. Behavioral Ecology and Sociobiology.

[B57-ejihpe-16-00044] Saks K., Hunt P., Leijen Ä., Lepp L. (2022). To stay or not to stay: An empirical model for predicting teacher persistence. British Journal of Educational Studies.

[B58-ejihpe-16-00044] Samala A. D., Rawas S., Criollo-C S., Bojic L., Prasetya F., Ranuharja F., Marta R. (2024). Emerging technologies for global education: A comprehensive exploration of trends, innovations, challenges, and future horizons. SN Computer Science.

[B59-ejihpe-16-00044] Sandoval-Obando E., Barros-Osorio C., Castellanos-Alvarenga L., Villalta Paucar M., Vega-Muñoz A. (2025). Generativity and psychological well-being in primary and secondary teachers: A systematic review. Societies.

[B60-ejihpe-16-00044] Sandoval-Obando E. E., Pareja-Arellano N., Hernández-Mosqueira C., Riquelme-Brevis H. (2023). What do we know about rural teaching identity? An exploratory study based on the generative-narrative approach. Journal of Pedagogy.

[B61-ejihpe-16-00044] Schaufeli W. (2021). Engaging leadership: How to promote work engagement?. Frontiers in Psychology.

[B62-ejihpe-16-00044] Schaufeli W., Bakker A. (2003). Utrecht work engagement scale.

[B63-ejihpe-16-00044] Schaufeli W. B. (2016). Heavy work investment, personality and organizational climate. Journal of Managerial Psychology.

[B64-ejihpe-16-00044] Schaufeli W. B., Bakker A. B., Van Rhenen W. (2009). How changes in job demands and resources predict burnout, work engagement, and sickness absenteeism. Journal of Organizational Behavior.

[B65-ejihpe-16-00044] Schaufeli W. B., Taris T. W., Bauer G. F., Hämmig O. (2014). A critical review of the job demands-resources model: Implications for improving work and health. Bridging occupational, organizational and public health.

[B66-ejihpe-16-00044] Schermelleh-Engel K., Moosbrugger H., Müller H. (2003). Evaluating the fit of structural equation models: Tests of significance and descriptive goodness-of-fit measures. Methods of Psychological Research.

[B67-ejihpe-16-00044] Sharif R. (2025). Beyond burnout: A counterintuitive analysis of how strain-based work-family interference strengthens affective, continuance, and normative commitment in Pakistan’s academia. Saudi Journal of Humanities and Social Sciences.

[B68-ejihpe-16-00044] Shu K. (2022). Teachers’ commitment and self-efficacy as predictors of work engagement and well-being. Frontiers in Psychology.

[B69-ejihpe-16-00044] Siddiqui S., Arif I., Hinduja P. (2023). Technostress: A catalyst to leave the teaching profession—A survey designed to measure technostress among teachers in Pakistan during COVID-19 pandemic. E-Learning and Digital Media.

[B70-ejihpe-16-00044] Siegrist J., Li J. (2016). Associations of extrinsic and intrinsic components of work stress with health: A systematic review of evidence on the effort-reward imbalance model. International Journal of Environmental Research and Public Health.

[B71-ejihpe-16-00044] Sobral F., Dias-Oliveira E., Morais C., Hodgson J. (2025). Blurred boundaries: Exploring the influence of work-life and life-work conflicts on university teachers’ health, work results, and willingness to teleworking. Frontiers in Education.

[B72-ejihpe-16-00044] Song C., Kawai R. (2023). Adaptive stratified sampling for structural reliability analysis. Structural Safety.

[B73-ejihpe-16-00044] Tabak F., Tziner A., Shkoler O., Rabenu E. (2021). The complexity of Heavy Work Investment (HWI): A conceptual integration and review of antecedents, dimensions, and outcomes. Sustainability.

[B74-ejihpe-16-00044] Timotheou S., Miliou O., Dimitriadis Y., Sobrino S. V., Giannoutsou N., Cachia R., Monés A. M., Ioannou A. (2023). Impacts of digital technologies on education and factors influencing schools’ digital capacity and transformation: A literature review. Education and Information Technologies.

[B75-ejihpe-16-00044] Tomczyk Ł., Majkut A. (2025). Integrating AI in education: An analysis of factors influencing the acceptance, concerns, attitudes, competencies and use of generative artificial intelligence among Polish teachers. Human Behavior and Emerging Technologies.

[B76-ejihpe-16-00044] Torres-Rivera A. D., Rendón Peña A. A., Díaz-Torres S. T., Díaz-Torres L. A. (2025). Ethical integration of AI and STEAM pedagogies in higher education: A sustainable learning model for society 5.0. Sustainability.

[B77-ejihpe-16-00044] Ubal Camacho M., Tambasco P., Martínez S., García Correa M. (2023). El impacto de la inteligencia artificial en la educación. Riesgos y potencialidades de la IA en el aula. RiiTE Revista Interuniversitaria De investigación En Tecnología Educativa.

[B78-ejihpe-16-00044] Uzuntiryaki-Kondakci E., Kirbulut Z. D., Sarici E., Oktay O. (2022). Emotion regulation as a mediator of the influence of science teacher emotions on teacher efficacy beliefs. Educational Studies.

[B79-ejihpe-16-00044] Van De Werfhorst H. G., Kessenich E., Geven S. (2022). The digital divide in online education: Inequality in digital readiness of students and schools. Computers and Education Open.

[B80-ejihpe-16-00044] Wang C., Chen X., Yu T., Liu Y., Jing Y. (2024). Education reform and change driven by digital technology: A bibliometric study from a global perspective. Humanities and Social Sciences Communications.

[B81-ejihpe-16-00044] Wang K., Ye Z., Li Z., Li S., Yuan X. (2025). Predicting teachers’ continuance intention to teach online: The role of technostress, intrapersonal and school environmental factors. Sage Open.

[B82-ejihpe-16-00044] Weber P. M., Kammerl R., Schiefner-Rohs M. (2025). What does digital well-being mean for school development? A theoretical review with perspectives on digital inequality. Education Sciences.

[B83-ejihpe-16-00044] Wettstein A., Schneider S., Jenni G., Holtforth M. G., Tschacher W., La Marca R. (2022). Association between workaholism, vital exhaustion, and hair cortisol concentrations among teachers: A longitudinal study testing the moderation effect of neuroticism. Frontiers in Psychology.

[B84-ejihpe-16-00044] Willermark S., Högberg K., Nilsson P. (2023). Exploring technostress in disruptive teaching practices. International Journal of Workplace Health Management.

[B85-ejihpe-16-00044] Xiao Y., Fathi J., Mohammaddokht F. (2022). Exploring a structural model of teaching enjoyment, teacher self-efficacy, and work engagement. Frontiers in Psychology.

[B86-ejihpe-16-00044] Ximénez M. C., García A. G. (2005). Comparison of maximum likelihood and unweighted least squares estimation methods in confirmatory factor analysis by Monte Carlo simulation. Psicothema.

[B87-ejihpe-16-00044] Xing Z. (2022). English as a foreign language teachers’ work engagement, burnout, and their professional identity. Frontiers in Psychology.

[B88-ejihpe-16-00044] Yan L., Suleman Abdullah Alwabel A., Mohamad U. H. (2025). AI-powered education: Transforming teacher-student interactions and advancing sustainable learning practices. European Journal of Education.

[B89-ejihpe-16-00044] Yang X., Zhu X., Chen D. (2023). Discourses regarding education governance in the digital age at K-12 level: Possibilities, risks, and strategies. Teaching and Teacher Education.

[B90-ejihpe-16-00044] Yuan H., Yan Z., Zhao Y., Lei J. (2025). The relationship of rural kindergarten teachers’ emotional intelligence and work engagement in China: The chain mediation role of emotional labor strategies and general self-efficacy. BMC Psychology.

[B91-ejihpe-16-00044] Zhai X. (2025). Transforming teachers’ roles and agencies in the era of generative AI: Perceptions, acceptance, knowledge, and practices. Journal of Science Education and Technology.

[B92-ejihpe-16-00044] Zhang A., Yang Y. (2021). Toward the association between EFL/ESL teachers’ work engagement and their students’ academic engagement. Frontiers in Psychology.

[B93-ejihpe-16-00044] Zhang H., Cao J. (2025). From digital disruption to mental health: The impact of AI-induced educational anxiety on teacher well-being in the era of smart education. BMC Public Health.

[B94-ejihpe-16-00044] Zhong Y., Rosli M. S. B. (2025). Multidimensional analysis of teachers’ technostress: Sources, impacts, and mitigation strategies. International Journal of Academic Research in Progressive Education and Development.

[B95-ejihpe-16-00044] Zorde O., Lapidot-Lefler N. (2025). Sustainable educational infrastructure: Professional learning communities as catalysts for lasting inclusive practices and human well-being. Sustainability.

